# Fotobiomodulación con oxígeno activo y lactoferrina en el tratamiento de osteonecrosis de la mandíbula asociada a medicamentos. Reporte de caso

**DOI:** 10.21142/2523-2754-1204-2024-223

**Published:** 2024-11-23

**Authors:** Carlos Sánchez Ramírez, Leixer de Santiago, Ana Bernotti, Patricia Moreno Garcés, Érica de Jesús, Alberto Miselli, Mariana Villarroel Dorrego

**Affiliations:** 1 Escuela de Odontologia, Universidad Jose Antonio Paez. Valencia, Venezuela. odcarlossanchez@gmail.com , ericadejesusdeleca@gmail.com Universidad José Antonio Páez Escuela de Odontologia Universidad Jose Antonio Paez Valencia Venezuela odcarlossanchez@gmail.com ericadejesusdeleca@gmail.com; 2 Centro Odontologico Bernotti Group. Caracas, Venezuela. bernottianaluisa@gmail.com, ladsh@hotmail.com, pattyrusia@gmail.com, dentalmis@gmail.com Centro Odontologico Bernotti Group Caracas Venezuela bernottianaluisa@gmail.com ladsh@hotmail.com pattyrusia@gmail.com dentalmis@gmail.com; 3 Escuela de Odontologia, Universidad Central de Venezuela. Caracas, Venezuela. mariana.villarroel@ucv.ve Universidad Central de Venezuela Escuela de Odontologia Universidad Central de Venezuela Caracas Venezuela mariana.villarroel@ucv.ve

**Keywords:** osteonecrosis de la mandíbula, fotobiomodulación, necrosis ósea, láser, cavidad bucal, Bluem, oxígeno activo, lactoferrina, jaw osteonecrosis, photobiomodulation, bone necrosis, laser, oral cavity, Blue®M, active oxygen, lactoferrin

## Abstract

La osteonecrosis de los maxilares asociada con medicamentos (MRONJ) afecta alrededor del 5% de los pacientes tratados con bisfosfonatos y otros fármacos similares. En el presente reporte de caso se describe el manejo de un caso de MRONJ utilizando terapia de fotobiomodulación (FBM) combinada con oxígeno activo y lactoferrina (Blue®M). Se trata de una paciente de 62 años con hipertensión, artritis reumatoide y osteoporosis tratada con Bonames®. Tras una exodoncia presentó hueso expuesto en el reborde alveolar del cuadrante III. Bajo consentimiento informado, se realizó una intervención quirúrgica mínimamente invasiva con instrumental pieza eléctrico (Piezotome®). El análisis histopatológico reveló tejido óseo necrótico con infiltrado inflamatorio crónico. El tratamiento incluyó Blue®M teeth&bone una vez al día, durante 30 días, aplicación tópica de gel Blue®M cada mañana, durante 15 días, y FBM con láser de diodo de 808 nm (Therapy-DMC®). Las sesiones de láser fueron diarias hasta el día 3, y luego dos veces por semana durante cuatro semanas. La paciente también realizó enjuagues bucales con Blue®M todas las noches por 30 días. Inicialmente, la paciente reportó un dolor de 3/10 según la escala visual análoga, que disminuyó a 0/10 al final del tratamiento. Clínicamente, se observó curación del tejido blando a los 30 días y a los 60 días, y en la tomografía se observó hueso esponjoso hiperdenso con cortical bien formada y sin alteraciones. Finalmente, este caso sugiere que la combinación de FBM con terapia tópica de oxígeno activo y lactoferrina, junto con una intervención quirúrgica mínimamente invasiva, es prometedora para el manejo de MRONJ.

## Abstract (en)

Medication-related osteonecrosis jaw (MRONJ) affects about 5% of patients treated with bisphosphonates and other similar drugs. This case report describes the management of a case of MRONJ using photobiomodulation therapy (FBM) combined with active oxygen and lactoferrin (Blue®M). This is a 62-year-old patient with hypertension, rheumatoid arthritis and osteoporosis treated with Bonames®. After extraction, she presented exposed bone in the alveolar ridge of quadrant III. Under informed consent, minimally invasive surgery was performed with electrical instruments (Piezotome®). Histopathological analysis revealed necrotic bone tissue with chronic inflammatory infiltrate. The treatment included Blue®M teeth&bone once a day for 30 days, topical application of Blue®M gel every morning for 15 days and FBM with 808nm diode laser (Therapy-DMC®). The laser sessions were daily until day 3, and then twice a week for four weeks. The patient also performed mouthwashes with Blue®M every night for 30 days. Initially, the patient reported pain of 3/10 on the visual analog scale, which decreased to 0/10 at the end of treatment. Clinically, soft tissue healing was observed at 30 days and 60 days and the CT scan showed hyperdense cancellous bone with well-formed cortical and no alterations. Finally, this case suggests that the combination of FBM with topical active oxygen therapy and lactoferrin, along with minimally invasive surgical intervention, holds promise for the management of MRONJ.

## INTRODUCCIÓN

La osteonecrosis de los maxilares asociada con medicamentos (MRONJ) es una reacción adversa que causa destrucción y necrosis ósea en la mandíbula y el maxilar, lo que afecta a pacientes tratados con fármacos antirresortivos o angiogénicos ^1^^-^^3^. Se caracteriza por la presencia de hueso expuesto o necrótico en la región maxilofacial durante más de ocho semanas y en ausencia de radioterapia o enfermedad metastásica ^4^.

La MRONJ resulta de la interacción entre fármacos. Los fármacos implicados incluyen inhibidores de la angiogénesis y bifosfonatos ^5^. Los inhibidores de la angiogénesis interfieren con la formación de nuevos vasos sanguíneos, lo que podría contribuir a la osteonecrosis al impedir el suministro vascular adecuado ^6^. Por otra parte, los bifosfonatos, indicados para tratar la osteoporosis y la osteopenia, inhiben la diferenciación y función de osteoclastos, lo cual reduce la resorción y remodelación ósea ^7^. Además, afectan negativamente a osteoblastos, fibroblastos y queratinocitos, al impedir la cicatrización y exponer el hueso. La alta tasa de remodelación ósea en los maxilares podría explicar su mayor susceptibilidad a la osteonecrosis ^8^. 

El tratamiento de la MRONJ es complejo y controvertido. Se priorizan enfoques conservadores y mínimamente invasivos para mejorar la calidad de vida del paciente mediante el control del dolor, la infección, y la minimización de la progresión de la necrosis ósea ^9^. Las estrategias terapéuticas dependen de la gravedad de la MRONJ, y abarcan desde una buena higiene bucal, el uso de enjuagues antisépticos y antibióticos, hasta la extirpación quirúrgica del hueso necrótico en casos severos, siendo esto último más eficaz a menudo, según diversos estudios ^10^^-^^12^.

Por tal motivo, la base de conocimientos y la experiencia para abordar la MRONJ continúa evolucionando y expandiéndose, lo que da lugar a terapéuticas coadyuvantes como la fotobiomodulación (PBM) con láser de diodo, que activa la cascada celular, produce trifosfato de adenosina mitocondrial y calcio intracelular, mejora el metabolismo energético celular y aumenta el suministro de sanguíneo, lo que genera mejores resultados en la cicatrización de los tejidos. Asimismo, esta terapia tiene propiedades analgésicas y antiinflamatorias, ya que reduce el dolor característico en estos pacientes, aumenta la liberación de beta-endorfinas y reduce la cantidad de citocinas inflamatorias, incluidas la bradicinina y las prostaglandinas, lo que conduce a una disminución de la inflamación, el edema y el dolor, así como de sus propiedades antimicrobianas ^13^. 

Del mismo modo, la terapia con gel de oxígeno activo y lactoferrina para potenciar la regeneración de los tejidos a corto plazo es una alternativa de tratamiento indicada por su acción regenerativa, analgésica, antiinflamatoria y antimicrobiana de amplio espectro. Destaca el potencial de la lactoferrina en la regulación de la actividad del sistema inmunitario, su acción bactericida bloqueando la adhesión y la entrada de patógenos en las células huésped, lo que inhibe las etapas iniciales de la infección, el aumento del crecimiento de fibroblastos, la síntesis de colágeno, el metabolismo celular, y la diferenciación y proliferación de células madre y osteoblastos. Asimismo, su papel en los procesos de regeneración de tejido óseo e inhibición de la apoptosis de osteoblastos y osteoclastogénesis ^14^^,^^15^. El objetivo de este reporte fue describir el manejo de un caso de MRONJ con terapia de FBM junto con oxígeno activo y lactoferrina (Blue®M).

## REPORTE DE CASO

**Figura 1:**
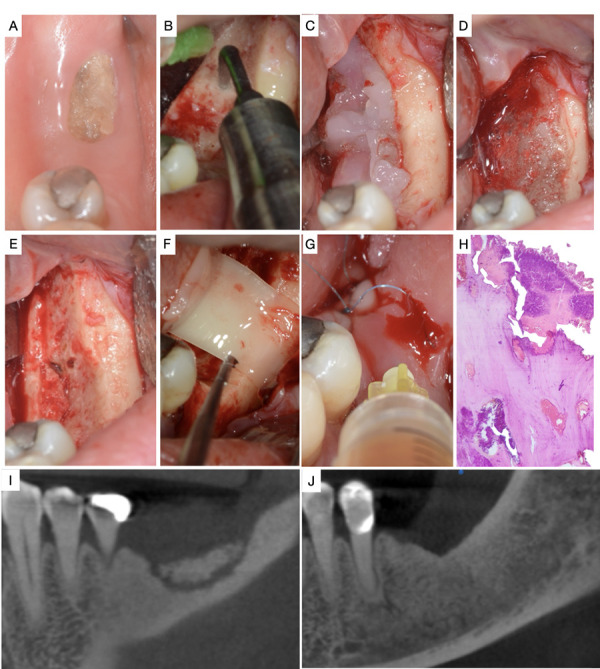
A) Hueso expuesto en el reborde alveolar del tercer cuadrante. B) Escisión del secuestro óseo con piezotome®. C) Aplicación de gel Blue®M. D) Acción a los 2 minutos de haber aplicado Blue®M de manera tópica. E) Hueso sangrante posterior al tratamiento con piezotome® y Blue®M. F) Colocación de membrana de PRF. G) Aplicación de líquido de PRF. H) Histopatología donde se observó tejido óseo con áreas necróticas, inflamación crónica extensa, focos prominentes de polimorfonucleares neutrófilos y abundantes colonias bacterianas. I) Tomografía computarizada donde se observa una imagen hiperdensa con bordes irregulares, compatible con secuestro óseo. J) Control tomográfico realizado a los 60 días, se observó hueso esponjoso hiperdenso con una cortical bien formada y sin alteraciones.

Se presenta el caso de una paciente femenina de 62 años con antecedentes de hipertensión, artritis reumatoide desde 2010 y osteoporosis desde 2015, tratada con ácido ibandrónico (Bonames® tabletas) de 150 mg, por vía oral, una vez al mes. Clínicamente, la paciente presentó hueso expuesto en el reborde alveolar del tercer cuadrante tras exodoncia en la zona (Figura 1A-G), acompañado de dolor y sangrado a la palpación en la periferia de la lesión.

El estudio histopatológico mostró tejido óseo con áreas necróticas, inflamación crónica extensa, focos prominentes de polimorfonucleares neutrófilos y abundantes colonias bacterianas (Figura 1H). La tomografía computarizada reveló una imagen hiperdensa con bordes irregulares, compatible con secuestro óseo (Figura 1I,J). A partir de las características clínicas, radiográficas e histopatológicas, se llegó al diagnóstico de osteonecrosis de la mandíbula relacionada con medicación (MRONJ) en estadio II, según la clasificación de la Asociación Americana de Cirujanos Orales y Maxilofaciales ^4^ (AAOMS). 

Para evaluar el dolor, se utilizó una escala visual analógica (EVA), donde 0 representa ausencia de dolor y 10 el mayor dolor posible. Antes de cualquier intervención, la paciente reportó una puntuación EVA de 3/10.

### Protocolo de tratamiento 

Previo consentimiento informado, se realizó una intervención quirúrgica mínimamente invasiva en la zona afectada. Bajo anestesia local, se procedió a realizar la debridación del tejido para la escisión del secuestro óseo con piezoeléctrico (piezotome®) (Figura 1B). La intervención continuó hasta que el hueso comenzó a sangrar, lo cual indicó la llegada a hueso sano. A continuación, se aplicó gel Blue®M, se lo dejó actuar durante 2 minutos y se lavó con solución fisiológica (Figuras 1C y 1D). Posteriormente, se colocó fibrina rica en plaquetas (PRF) ^16^, y se finalizó con puntos simples usando sutura de nailon no absorbible 5/0 (ARIZI®).

Se indicaron los siguientes cuidados posoperatorios, antibioticoterapia con amoxicilina más ácido clavulánico (875+125 mg) una vez al día durante 7 días, e ibuprofeno (400 mg) cada 8 horas por 3 días, solo en caso de dolor. Además, se prescribió una tableta diaria de Teeth&Bone fórmula Blue®M durante 30 días. Se recomendó no realizar enjuagues bucales durante las primeras 72 horas; después de este periodo, se indicó el uso de enjuague bucal Blue®M una vez al día realizando buches retentivos por 60 seg. En el posoperatorio inmediato, se realizó terapia tópica con gel Blue®M sobre la herida, dejándolo actuar durante 2 minutos, y se continuó su aplicación de forma ambulatoria todas las mañanas durante 15 días.

**Figura 2:**
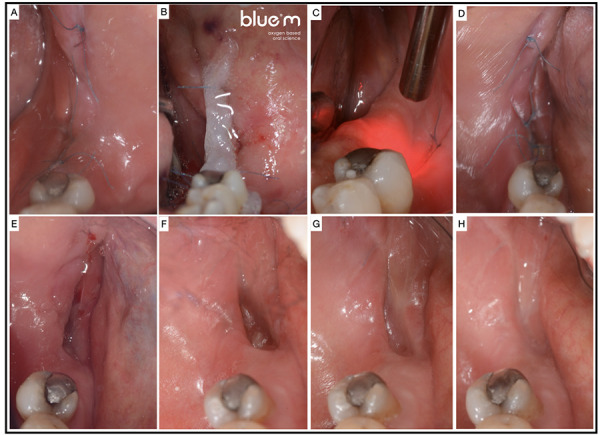
A) Puntos simples usando sutura de nylon no absorbible 5/0 (ARIZI®). B) Aplicación de gel Blue®M. C) Terapia de FBM utilizando un equipo de láser de diodo dual 650-808 (modelo Therapy EC DMC®). D) Cicatrización al día 7. E) Cicatrización al día 15. F) Cicatrización al día 25. G) Cicatrización al día 30. H) Cicatrización al día 60.

Se implementó terapia de FBM utilizando un equipo de láser de diodo dual 650-808 (modelo Therapy EC DMC®), con una potencia de 100 mW. Se seleccionó una longitud de onda infrarroja de 808 nm con emisión continua para la aplicación, con un área de irradiación de 0,0984 cm². La aplicación del láser se llevó a cabo con contacto perpendicular a la zona, de forma puntual y siguiendo el protocolo descrito en la literatura^17^, con una distancia de 1 cm entre puntos a lo largo del trayecto de la herida (Figura 2). La terapia de FBM se realizó en sesiones diarias de 4 joules por cada 2 cm² hasta el día 3, y luego dos veces por semana durante 4 semanas.

La dosificación de la densidad de energía (Tabla 1) se calculó según la fórmula dE = E/A. Como resultado, se obtuvieron las siguientes densidades de energía utilizadas:

dE = E/A dE = Densidad de energía E = energía (4J) A = área (0,0984 cm^2^)

dE = 4J/ 0,0984cm^2^ = 40,64J/cm^2^

**Tabla 1:** Parámetros usados láser de diodo dual 650-808 (modelo Therapy EC DMC®)

Especificaciones	
Diámetro (mm)	3,54
Área (cm^2^)	0,0984
Energía (julios)	4
Densidad de energía	40,64

### Evolución

Se realizaron controles diarios durante los primeros 4 días, y posteriormente a los 7, 15, 25, 30 y 60 días. Al inicio del tratamiento, la paciente reportó un dolor de 3/10, según EVA, el cual disminuyó a 0/10 al final del tratamiento. Clínicamente, se observó una curación completa del tejido blando a los 30 días de iniciado el tratamiento (Figura 2). En el control tomográfico realizado a los 60 días, se observó hueso esponjoso hiperdenso con una cortical bien formada y sin alteraciones (Figura 1H). La paciente informó estar satisfecha con el tratamiento, destacando la ausencia de dolor y una mejora significativa en la capacidad de deglución y la calidad de vida.

## DISCUSIÓN

Desde su primera descripción en la literatura en 2003, la MRONJ ha sido objeto de numerosos estudios que han identificado una amplia gama de fármacos implicados en su desarrollo, así como los efectos adversos de los bisfosfonatos en diversas líneas celulares. A lo largo de los años, se han publicado múltiples investigaciones que abordan las complicaciones, los signos y síntomas clínicos, las características radiográficas, la posible fisiopatología y el tratamiento de esta patología. No obstante, en cuanto a las estrategias terapéuticas, se han propuesto diversos protocolos y métodos complementarios para el manejo de la MRONJ ^18^.

Diversas series de casos han documentado la aplicación de la PBM en el tratamiento de pacientes con MRONJ. Los efectos positivos de la PBM pueden lograrse mediante la aplicación de diferentes longitudes de onda dentro de una ventana terapéutica de 1 a 500 mw, para efectos de esta investigación se utilizó una longitud de onda de 100 mw encontrándose dentro de los parámetros recomendados en la literatura, reportando beneficios significativos para pacientes con diagnóstico de MRONJ. La efectividad de la terapia con láser de baja intensidad en la promoción del crecimiento celular en diferentes tejidos de la cavidad oral es bien conocida y se utiliza para acelerar la cicatrización mediante el aumento de la actividad mitótica y los cambios en la síntesis de colágeno característicos de esta terapia ^19^. Sun *et al*. ^20^ subrayan la importancia de aplicar la terapia a 1 cm de distancia de la herida. Además, Reis *et al*. ^17^, en una revisión sistemática, llegaron a la conclusión de que la longitud de onda entre los 830 nm ofrece mejores resultados, así como el modo de emisión continuo para la MRONJ, lo que concuerda con los obtenidos en esta investigación.

Cabe destacar que la dosificación precisa de la FBM es fundamental para garantizar la eficacia del tratamiento. Por lo tanto, es necesario calcular meticulosamente la densidad de energía en relación con el área irradiada. Este aspecto representa uno de los principales desafíos al comparar los estudios revisados, ya que no todos realizan este cálculo de manera precisa, lo que puede generar parámetros incorrectos y resultados impredecibles. Los estudios revisados mostraron que parámetros similares resultaron beneficiosos. Monteiro *et al*. ^21^ evaluaron un caso de MRONJ en el maxilar, en el que se aplicaron 5 sesiones de FBM con un láser de diodo de 635 nm y una densidad de energía de 10 J/cm² en la zona afectada. Los resultados mostraron un alivio del dolor en la primera sesión y la integridad del tejido fue confirmada clínica y radiográficamente un mes después. Al control a los 6 meses, no hubo recidiva. Concluyeron que la FBM es un tratamiento no invasivo que facilita la cicatrización y reparación exitosa de los tejidos. 

Otros autores ^22^ concluyeron que, a una densidad de energía de 60 J/cm² y una longitud de onda de 830 nm, los fibroblastos humanos sanos mostraron una mejor proliferación celular y síntesis de colágeno. Jamalpour *et al*. ^23^ utilizaron una densidad de energía de 17,85 J/cm², logrando una curación óptima de heridas y regeneración ósea en las lesiones de MRONJ. Es relevante señalar que la densidad de energía utilizada en este estudio estuvo dentro del rango máximo-mínimo reportado en la literatura. Por lo tanto, se concluye que las densidades de energía ideales oscilan entre 17,85 J/cm² y 60 J/cm². También es importante señalar que, en estos estudios, el área de irradiación fue menor que la del presente estudio, lo cual sugiere una posible utilización de una energía total mayor, además de proporcionar un alivio inmediato del dolor y la necesidad de llegar a tejidos más profundos.

De manera similar, Tenore *et al*. ^24^ compararon tres protocolos de tratamiento en pacientes con MRONJ. El grupo 1 fue tratado con antibioticoterapia, cirugía, fibrina rica en plaquetas y leucocitos (L-PRF) y PBM; el grupo 2 con terapia antibiótica y cirugía; y el grupo 3 con antibioticoterapia y PBM. Los resultados indicaron que el mejor protocolo fue el que combinó cirugía con L-PRF y PBM, lo que concuerda con otros estudios ^25^^-^^27^, lo que respalda el enfoque utilizado en la presente investigación.

En relación con la suplementación con oxígeno, se ha demostrado que esta contribuye a la muerte oxidativa de las bacterias, estimula la angiogénesis, acelera la formación de la matriz extracelular, aumenta la proliferación de fibroblastos y la deposición de colágeno, lo que facilita una cicatrización más rápida ^28^. El gel oral Blue®M, un producto que libera oxígeno y lactoferrina presenta altas propiedades antimicrobianas, ha mostrado eficacia en estudios *in vitro*, como Deliberador *et al*. ^28^ quienes demostraron que este gel inhibe el crecimiento de *Porphyromonas gingivalis*, lo que sugiere que su acción bacteriana de amplio espectro es favorable en casos de MRONJ, al reducir la persistencia y gravedad de la lesión.

Mattei *et al*. ^29^ demostraron en 2021 que el enjuague bucal con Blue®M reduce el dolor posoperatorio y los signos clínicos de inflamación en heridas de la mucosa oral. Estos resultados respaldan los hallazgos de la presente investigación, en la que se utilizó este producto en múltiples presentaciones. La paciente refirió alivio del dolor y una óptima microflora bucal para la cicatrización adecuada de los tejidos, por lo cual se propone este producto como coadyuvante en el tratamiento de la MRONJ. Deliberador *et al*. ^30^ evaluaron el uso de un gel tópico de liberación de oxígeno y lactoferrina en la cicatrización de heridas cutáneas estandarizadas en ratones. Los resultados mostraron una mayor angiogénesis y mejor formación de fibras de colágeno, junto con niveles significativamente mayores de TNF-α y VEGF en el grupo tratado con el gel de oxígeno. Este estudio apoya los beneficios de la terapia con oxígeno y lactoferrina en la cicatrización, lo cual coincide con los resultados observados en la presente investigación, por lo que podría proponerse como terapia coadyuvante en la higiene de los pacientes con diagnóstico de MRONJ gracias a sus propiedades ya mencionadas.

En el presente estudio, se ha analizado la influencia de la PBM, junto con la terapia de oxígeno activo y lactoferrina en sus diversas presentaciones, y se obtuvieron resultados positivos que incluyeron la cicatrización exitosa de los tejidos involucrados, el alivio inmediato de los síntomas de dolor y mejoras en la calidad de vida del paciente. Estos datos sugieren la utilización de la PBM y la terapia de oxígeno activo con lactoferrina como coadyuvantes eficaces y seguros en el tratamiento de la MRONJ, por lo que resulta una alternativa no invasiva que ofrece importantes beneficios; sin embargo, debido al alto costo de la PBM y la terapia de oxígeno activo, es un tratamiento no asequible para todos los pacientes, factor que debe ser considerado. Asimismo, se necesitan más estudios para afirmar que dicho protocolo es eficaz para el manejo de la MRONJ, considerando que dicha investigación estableció el reporte de un solo caso.

## CONCLUSIÓN

El uso de la PBM junto con la terapia de oxígeno activo y lactoferrina (Blue®M) como coadyuvantes en el tratamiento de la MRONJ ofrecen resultados positivos al mejorar la calidad de vida del paciente, la cicatrización de los tejidos involucrados y la disminución del dolor, sin efectos secundarios ni recidivas.

## Financiamiento:

Autofinanciado.
